# Treatment of Plane Warts with a Low-Dose Oral Isotretinoin

**DOI:** 10.5402/2012/163929

**Published:** 2012-12-12

**Authors:** Hayder R. Al-Hamamy, Husam Ali Salman, Nawar A. Abdulsattar

**Affiliations:** ^1^Department of Dermatology and Venereology, College of Medicine, University of Baghdad, Medical Collection Office, P.O. Box 61211, Baghdad 12114, Iraq; ^2^Department of Dermatology and Venereology, Baghdad Teaching Hospital, Medical City, Baghdad, Iraq

## Abstract

*Objective*. To assess the efficacy of a low-dose oral isotretinoin in the treatment of plane warts. *Patients and Methods*. Thirty-one patients with recalcitrant facial plane warts were enrolled. A cumulative dose of 30 mg/kg for two months of treatment was calculated; this was equal to a mean of 0.5 mg/kg/day. Each patient was seen every two weeks during the treatment period. Response to treatment was either complete or no response. Patients with complete response were followed up monthly for four months to record the relapse rate. *Results*. Twenty-six patients completed the study; their ages range from 5 to 35 with a mean ± SD 15.28 ± 8.51 years. Fifteen (57.69%) patients were females and eleven (42.30%) were males. Nineteen (73.07%) patients showed complete response and seven (26.92%) patients showed no response at the end of two months of therapy. The difference was statistically significant; *P* value <0.0001. Fifteen (78.94%) out of nineteen patients, who had complete response, were still free from warts at the end of four-month followup. *Conclusion*. Oral isotretinoin is effective in the treatment of recalcitrant facial plane warts.

## 1. Introduction

Plane warts are smooth, flat, or slightly elevated and are usually skin coloured or greyish yellow but may be pigmented. They are round or polygonal in shape and vary in size from 1 to 5 mm or more in diameter. They usually affect the face and the dorsa of the hands [1]. Many modalities of therapy had been used with variable success, for example, topical retinoic acid [2], imiquimod [3], and 5-fluorouracil [4]. 

Systemic isotretinoin has been used to treat severe acne vulgaris. However, isotretinoin also represents a potentially useful choice in many dermatologic diseases such as psoriasis, pityriasis rubra pilaris, condylomata acuminata, skin cancers, and rosacea [5].

The aim of the present study is to evaluate the effectiveness of a low-dose oral isotretinoin in the treatment of plane warts.

## 2. Patients and Methods

This was an open therapeutic trial conducted at the department of Dermatology and Venereology, Baghdad Teaching hospital from August 2009 to March 2011.

Thirty-one patients with facial plane warts were included in the study. All cases were resistant to other forms of therapy and were without any treatment for at least two months before beginning the study. 

All female patients enrolled in the study were unmarried. Children under 5 years and pregnant women were excluded from the study. 

 A full interrogation and explanation about the nature of the disease and the possible side effects of the treatment were performed to each patient and/or the patient's parent. Then a formal consent was obtained from each patient or his/her parent.

The study was approved by the ethical committee.

Each patient was treated with oral isotretinoin (Retane) capsules from Asia Pharmaceutical Industries, Syria. For each patient a cumulative dose of 30 mg/Kg for two-month period of treatment was calculated. Then the number of isotretinoin capsules (10 and/or 20 mg) required for the whole treatment period was calculated accordingly. The number of capsules was divided to 60 days of treatment. This was equal to a mean dose of 0.5 mg/kg/day.

Each patient was instructed to use white petrolatum jelly (Vaseline) as needed to reduce lip dryness.

Each patient was seen every two weeks during the treatment period, to assess the response to treatment and to report the side effects. The response was either any of the following.

Complete response: when there is complete disappearance of warts. 

No response: when there is no or partial improvements.

For each patient the lipid profile, liver function test, and complete blood count were performed before treatment and every four weeks during the treatment period.

**Followup**

Patients with complete response were followed up monthly for four months to record the relapse rate.

Descriptive and analytical statistics were done by graph pad software. Chi square was used to compare the results; *P* value of less than 0.05 was considered to be statistically significant.

## 3. Results

Twenty-six patients completed the study; their ages range from 5 to 35 with a mean ± SD 15.28 ± 8.51 years. Fifteen (57.69%) patients were females, and 11 (42.30%) were males. Five patients defaulted from the study during the treatment period for unknown reasons.

The duration of warts ranged from 8–48 with a mean ± SD 18.69 ± 9.19 months. Nineteen (73.07%) patients showed complete response and seven (26.92%) patients showed no response at the end of two months therapy. The difference was statistically significant; *P*  value < 0.0001.

All side effects were minimal and did not require discontinuation of therapy (Table 1).

All the laboratory investigations before and during the treatment were normal for all patients.

**Followup**

**Figure 1:**
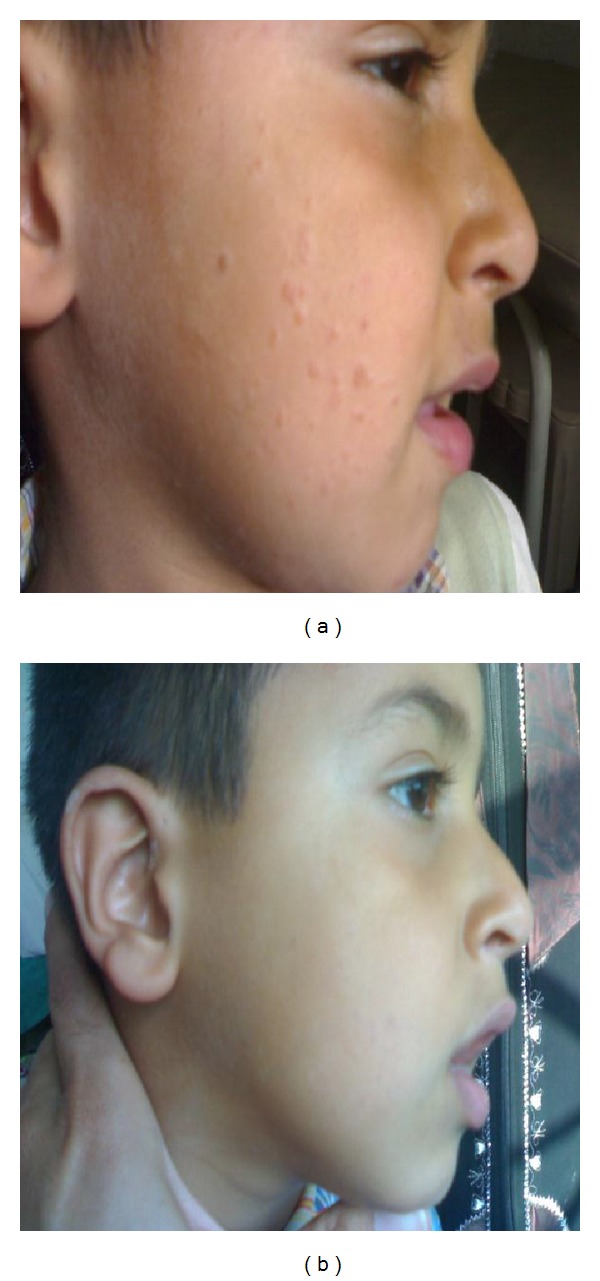
(a) A nine-year-old male with plane warts for 48 months duration before treatment. (b) The same patient after treatment and 3 months of followup.

Fifteen (78.94%) out of 19 patients, who had complete response, were still free from warts at the end of four months followup (see Figure 1).

## 4. Discussion

 Flat warts are a common therapeutic problem. Disseminated infection on the face is, even in the immunocompetent host, a challenge [6].

 Conventional therapies for human papillomavirus (HPV) infection are often associated with unsatisfactory response rates and high recurrence rates. The use of a systemic agent may more effectively control the virus [7].

 Reviewing the literatures revealed that few studies that had been published showed the effectiveness of systemic isotretinoin for condylomata accuminata [5], and etretinate has been helpful for treating hyperkeratotic warts in immunosuppressed patients [8].

To our knowledge, the present study is the first that assesses the efficacy of an oral retinoid to treat plane warts.

The standard cumulative dose of oral isotretinoin for acne vulgaris is 120 mg/kg; this equals to 1 mg/kg/day for four months [9]. However, this potentially high dose is associated with a high profile of side effects [10].

Many studies in the last years tried a low dose, for example, 38.4 mg/kg cumulative dose of isotretinoin in acne [10].

Other study revealed that the efficacy and relapse rates of low-dose isotretinoin (0.5 mg/kg/day) in mild to moderate grades of acne are comparable with the standard regimen (1 mg/kg/day) [11].

For the above reasons and to reduce the side effects, we used a low cumulative dose of isotretinoin (30 mg/kg). 

Premature closure of epiphyses, although more common in children, is rare and occurred at higher doses [12]. Cases that had been reported with this problem are few and on long duration of therapy, for example, a 6-year-old girl with short stature which developed following the administration of 13-*cis*-retinoic acid (isotretinoin) for 40 months [13]; other case is a boy with epidermolytic hyperkeratosis who was treated systemically for 4 and half years with 13-*cis*-retinoic acid. At the age of 10 and half years, he developed pain in his right knee and radiographic evidence of partial closure of the proximal epiphysis of the right tibia [14].

Comparing the results of the present study to topical imiquimod, the latter is a unique topical therapeutic agent useful in the treatment of external genital and perianal warts (condyloma acuminata) in adults. It was used in individual cases for plane warts, and it also was expensive [3].

The results of the present study were comparable to 5-fluorouracil ointment, but most patients treated with the latter showed hyperpigmentation, and many of them showed erosions [4].

Pulsed dye laser treatment is a relatively effective treatment for plane warts with a clearance rate 67.6% [15]. The result was slightly lower than the present study (73.07%). Also pulsed dye laser needs many sessions, not available widely and expensive. 

Flat warts present a special therapeutic problem. Their duration may be lengthy and may be very resistant to treatment [12]. Therefore, oral isotretinoin seems to be most efficacious therapy for recalcitrant plane warts when compared with the previous studies with acceptable cost, wide availability, and it seems to be safe in children with mild and reversible side effects and no serious problems when used for a relatively short period and with a low cumulative dose.

We think that using a cumulative dose less than 30 mg/kg is of poor value, because of the expected less response and a high relapse rates. However, patients who cannot tolerate the 0.5 mg/kg/day regimen can be treated with 0.25 mg/kg/day for four months to reach the same 30 mg/kg cumulative dose with better tolerance to side effects and with a possible same response and relapse rates.

We think also that increasing the cumulative dose to 60 mg/kg for four months will result in an increase of the response and a decrease of the relapse rates, and we recommend further study using such a regimen to treat recalcitrant facial plane warts in adults who can tolerate more side effects. 

## Figures and Tables

**Table 1:** Side effects of oral isotretinoin.

Side effect	Number of patients	Percentage
Dry lips	26	100
Dry skin	20	76.92
epistaxis	2	7.69
Dry nose	8	30.76
headache	1	3.84
